# Recent Reports on Bioactive Compounds from Marine Cyanobacteria in Relation to Human Health Applications

**DOI:** 10.3390/life13061411

**Published:** 2023-06-19

**Authors:** R. M. T. D. Perera, K. H. I. N. M. Herath, K. K. Asanka Sanjeewa, Thilina U. Jayawardena

**Affiliations:** 1 Department of Biosystems Technology, Faculty of Technology, University of Sri Jayewardenepura, Pitipana 10206, Sri Lanka; 2 Department of Bio-Systems Engineering, Faculty of Agriculture and Plantation Management, Wayamba University of Sri Lanka, Makandura 60170, Sri Lanka; 3 Department of Chemistry, Biochemistry and Physics, Université du Québec à Trois-Rivières, Trois-Rivières, QC G8Z 4M3, Canada

**Keywords:** marine cyanobacteria, bioactive properties, bioactive compounds

## Abstract

The ocean is a valuable natural resource that contains numerous biologically active compounds with various bioactivities. The marine environment comprises unexplored sources that can be utilized to isolate novel compounds with bioactive properties. Marine cyanobacteria are an excellent source of bioactive compounds that have applications in human health, biofuel, cosmetics, and bioremediation. These cyanobacteria exhibit bioactive properties such as anti-inflammatory, anti-cancer, anti-bacterial, anti-parasitic, anti-diabetic, anti-viral, antioxidant, anti-aging, and anti-obesity effects, making them promising candidates for drug development. In recent decades, researchers have focused on isolating novel bioactive compounds from different marine cyanobacteria species for the development of therapeutics for various diseases that affect human health. This review provides an update on recent studies that explore the bioactive properties of marine cyanobacteria, with a particular focus on their potential use in human health applications.

## 1. Introduction

Cyanobacteria, also referred to as blue–green algae are believed to be one of the oldest organisms on Earth, which they have been inhabited throughout the last few billion years. Cyanobacteria are photoautotrophic, Gram-negative bacteria that can thrive in various environments including freshwater, marine, and soil. Although cyanobacteria have similar origins and basic anatomical features to bacteria, their ecological, biological, and physical characteristics are quite distinct and different from bacteria. The ability to harvest solar energy and perform photosynthesis through chlorophyll-a by fixing CO_2_ and generating O_2_ makes the cyanobacteria the largest photosynthetic prokaryotes [1]. Marine cyanobacteria can be divided into two large ecological groups: planktonic cyanobacteria and benthic cyanobacteria. Planktonic cyanobacteria float freely in water columns and can regulate their depth using the buoyancy regulation and production of aero-topes containing gas vesicles. The marine cyanobacterial genera *Prochlorococcus*, *Synechococcus*, *Synechocytis*, and *Cynobium* are abundantly found in marine planktonic communities. Benthic cyanobacteria possess unique gliding motility which aids them in contacting with solid surfaces like sediments, rocks, stones, algae, and aquatic plants [1]. The marine cyanobacterial genus *Lyngbya* can be found abundantly in benthic communities worldwide and it forms dense and widespread benthic cyanobacterial mats.

Cyanobacteria have vast distribution and are frequently found in aquatic and terrestrial environments as well as extreme environments with high salinity and high and low temperatures, as they have survival mechanisms to thrive in those extreme environmental conditions for a long period. Those survival mechanisms include the production of secondary metabolites with antioxidant, photoprotective, moisturizing, allelopathic, and toxic properties, which include pigments, polysaccharides, fatty acids, and peptides. These bioactive compounds found in cyanobacteria have great potential to be used in the formation or production of medicinal drugs. Cyanobacteria are not only a source of important pharmacological molecules, but they are also used for various purposes such as for food additives, biofuel production, cosmetics, animal feed, coloring dyes, wastewater treatment, and biopolymer production [2]. Cyanobacterial pigments have commercial value as natural colorants and dyes for food coloring and other purposes. In addition, cyanobacteria proved to be effective in removing heavy metals from industrial wastewater and for the bioremediation of aquatic habitats. These versatile organisms have multiple applications beyond just pharmaceuticals and can be utilized for various industrial and environmental purposes [3]. Marine cyanobacteria such as *Cylindrospermum* sp., *Phormidium* sp., *Pseudanabaena* sp., and *Spirulina* sp. were shown to be effective in the bioremediation of wastewater [4,5]. Marine cyanobacteria have their potential in the production of animal feed. *Spirulina platensis*, *Schizochytrium* sp., and *Arthrospira maxima* were reported to be used in animal feed production [6].

The bioactive compounds isolated from cyanobacteria consist of compounds with anti-inflammatory [7], anti-cancer [8], anti-bacterial [9], anti-parasitic [10], anti-diabetic [11], anti-viral [12], antioxidant [13], anti-aging [14,15], anti-obesity [16], hepatoprotective [17], immunomodulatory [18], photoprotective [19], and neuroprotective [20] activities [2]. So far, more than 2000 secondary metabolites have been identified from cyanobacteria, including marine cyanobacteria species like *Moorea*, *Lyngbya*, and *Okeania* spp. [21].

Global morbidity and mortality have been increasing during the past few decades. According to the world health organization, 7 of the 10 leading causes of death in 2019 were non-communicable diseases like cancers, Alzheimer’s disease, and diabetes mellitus [22]. Currently, synthetic drugs are being used to treat these diseases; however, numerous negative impacts are associated with these synthetic drugs such as toxicity, carcinogenicity, multi-drug resistivity, etc. Therefore, researchers are now focusing on drug discovery from natural resources to discover newer and more effective drug compounds to treat deadly human diseases.

Since the marine environment covers over 70% of the Earth’s surface, it is widely recognized as an untapped and highly promising source for the isolation of bioactive compounds [23]. However, though the isolation of bioactive compounds started from marine macroalgae, recent studies focused on the production and isolation of bioactive compounds and therapeutics from marine microalgae (cyanobacteria), as they are easier to cultivate and have short cultivation times [24]. Additionally, some research studies were conducted manipulating growth conditions by applying various forms of stress to the cells to promote the production of cyanobacteria biomass with valuable bioactive compounds [25]. Being a potential source for several bioactive compounds, marine cyanobacteria have shown potential applications in human welfare and medicine. There have been many studies investigating the pharmacological properties associated with marine cyanobacteria and their bioactive compounds.

**Figure 1:**
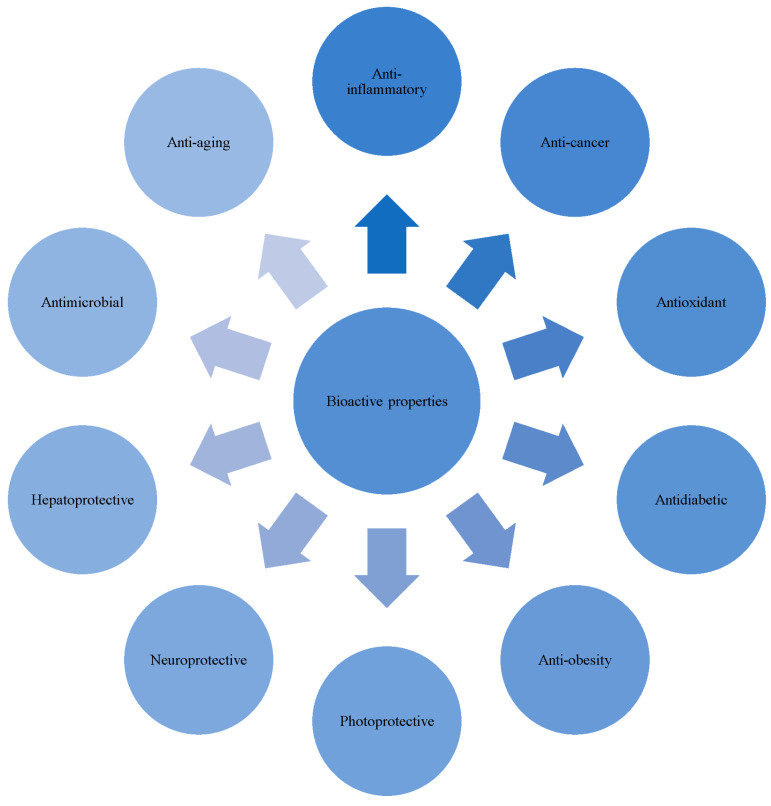
Bioactive properties from marine cyanobacteria in relation to human health applications.

Despite many bioactive properties being identified from marine cyanobacteria, in this review, the authors mainly focus on the major findings showing the recent bioactive properties (Figure 1) reported from various marine cyanobacterium species in relation to human health applications. Moreover, in this review the authors highlight the pharmacologically important bioactive compounds of marine cyanobacteria and their structure, as well as their bioactivity effects on various cell lines.

## 2. Materials and Methods

For the present study, the authors conducted a literature search from 2018 onwards using the databases Elsevier-Science Direct, PubMed, Google Scholar, Biomed Central, and Academia. The search included the keywords ‘Cyanobacteria’ and ‘Marine cyanobacteria’, cross-referenced with the keywords ‘Bioactive properties’, ‘Bioactive compounds’, ‘Antioxidant’, ‘Anti-inflammation’, ‘Anti-diabetic’, ‘Anti-obesity’, ‘Antimicrobial’, ‘Neuroprotective’, ‘Anti-aging’, ‘Photoprotective’, ‘Anticancer’, and ‘Hepatoprotective’. The references found in the search were then reviewed for details on the in vivo and in vitro assays used to test the bioactive properties of various cyanobacterium extracts or compounds.

## 3. Bioactive Properties Reported from Marine Cyanobacteria

### 3.1. Anti-Inflammatory Properties

Inflammation is the natural physiological reaction of the immune system to tissue damage that happens when the human body attempts to deal with different pathogens, toxic compounds, and damaged cells [26]. Inflammation can cause acute and chronic reactions in various organs, resulting in tissue injuries and immune-mediated diseases. *Spirulina* spp. contains Sulphoquinovosyl diacylglycerol (SQDG), a natural sulphoglycolipid molecule with anti-inflammatory, anti-viral, and anti-tumor properties. The Irish marine cyanobacterium *Spirulina subsalsa* is a rich source of glycolipids, including SQDG, monogalactosylodiglycerides (MGDG), and glycosphingolipids (cerebrosides and ceramides). It also contains phospholipids such as phosphatidylcholine (PC) and phosphatidylethanolamine (PE) molecules. According to Shiels et al. [7], the glycolipid and phospholipid fractions of *S. subsalsa* exhibited strong anti-inflammatory properties and *S. subsalsa* can be used as a promising candidate for developing novel health supplements with cardiovascular health benefits.

The bioactive peptides (MW < 3 kDa fractions) isolated from *Synechococcus* sp. showed anti-inflammatory properties by reducing the gene expression levels of the pro-inflammatory cytokines iNOS, TNF-α, COX-2, and IL-6 in LPS-stimulated RAW 264.7 macrophage cells. Therefore, this study suggested the possible application of the isolated peptides to develop natural anti-inflammatory drugs. A study by Lopes et al. [27], showed that the marine cyanobacterium *Leptolyngbya-like* sp. LEGE13412 reduced the nitric oxide (NO) in RAW 264.7 cell culture medium, making it a potential candidate for the treatment of psoriasis. A study on screening for polar lipids and the antioxidant and anti-inflammatory activities of *Gloeothece* sp. lipid extract with the concentration of 10 µg mL^−1^ found that it displayed anti-inflammatory activity by the inhibition of the conversion of arachidonic acid to prostaglandin H2 (PGH2) through the inhibition of COX-2 by about 58% [28]. According to a study conducted by Rai et al. [29], (9S, E)-8-ethyl-9-methylnonadec-6-en-3-one (EME) extracted from *Lyngbya* sp. demonstrated significant downregulation of the gene expressions of COX-2, TNF-α, iNOS, NF-kβ, and IL-1β in RAW 264.7 cells induced with Lipopolysaccharide (LPS) and treated with EME and aminated mesoporous silica nanoparticles. Additionally, Kirk et al. [30] isolated a cyanobacterial metabolite from *Trichodesmium thiebautii* that lowered the levels of TNF-α, IL-16, soluble TLR2, and nitric oxide species (NOS) in cell culture media. The study highlighted Unnarmicin D as the lead molecule for development of a drug for moderate chronic pain by reducing neuroinflammation. Malynglamide is another type of bioactive molecule present in marine cyanobacteria which exhibits anti-inflammatory properties. Malynglamide F was isolated from the marine cyanobacterium *Lyngbya majuscule* and was subjected to screening for its anti-inflammatory activity using rat models. The data obtained from this study demonstrated that Malynglamide F was capable of downregulating the levels of IL-6 and TNF-α in inflammation-induced rat models with carrageenan, formalin, xylene, AA, and cotton pellets. Downregulation of IL-6 and TNF-α cytokines indicates the potential of using Malynglamide F as an anti-inflammatory agent [31].

### 3.2. Anticancer Properties

Cancer is one of the most vital healthcare challenges in the world. Cancer is a heterogeneous disease that leads to the growth of abnormal cells that spread to other parts of the body. Chemotherapy is the most commonly used cancer treatment, which involves preventing cancer cells from dividing and growing. Since conventional chemotherapy drugs can have severe side effects, the use of natural anticancer products is gaining more attention at present. More than 50% of marine cyanobacteria produce bioactive compounds. The bioactive compounds produced by marine cyanobacteria are a rich source of compounds with potential anticancer effects which are capable of killing the cancer cells by affecting the cell signaling through activation of protein kinase-C family members or inducing apoptotic death [32].

In a recent study, Cai et al. [33] reported the anticancer activity of two cyclic lipopeptides, laxaphycins B4 (1) and A2, isolated from the marine cyanobacterium *Hormothamnion enteromorphoids*, which were evaluated using an MTT assay against human colon cancer HCT116 cells. According to the authors, laxaphycin B4 was found to possess anticancer activity in HCT116 cells with an IC_50_ value of 1.7 μM while laxaphycin A2 showed weak anticancer activity with an IC_50_ value of 2 μM. In addition, the anticancer properties of 62 cyanobacteria isolates from Brazil against acute myeloid leukemia (AML) cancer cell lines were evaluated by Shishido et al. [34]. The authors found that extracts from 39 strains resulted in cell death in AML cancer cell lines. Caldoramide, a formal pentapeptide metabolite of the marine cyanobacterium *Caldora penicillata*, was tested on the human colon adenocarcinoma cell line HT-29 (Table 1), the human colon carcinoma cell line HCT116, and the human breast adenocarcinoma cell line MCF-7 using an MTT assay [35]. Phycocyanin, a large, water-soluble phycobiliprotein extracted from the marine cyanobacterium *Spirulina platensis*, was tested against the Vero cell lines and Hep-G2 (liver cancer) cell lines using MTT assay. The maximum anticancer activity was observed at 500 μg/mL and the lowest anticancer activity was observed at the concentration of 100 μg/mL against Hep-G2 cell lines [11]. Fayyad et al. [36], also reported the anticancer activity of the hot methanolic extract of the cyanobacterium *S. platensis* against L20B and MCF-7 human cancer cell lines. According to the authors, the methanolic extract of *S. platensis* was found to possess antiproliferative properties against the L20B and MCF-7 cell lines after 24 h of incubation. Furthermore, methanol extract from the marine cyanobacterium *Nostoc* sp. N42 and *Fischerella* sp. S29 was tested against a human liver cancer cell line (HepG2) and human non-small-cell lung carcinoma (A-549) using an MTT assay. According to the authors, *Fischerella* sp. showed anticancer activity with an IC_50_ of 254.51 μg mL^−1^ against the human liver cancer cell line and an IC_50_ of 171.74 μg mL^−1^ against the human non-small-cell lung carcinoma cell line. *Nostoc* sp. N42 showed anticancer activity with an IC_50_ of 583.1 μg mL^−1^ against HepG2 and an IC_50_ of 792.17 μg mL^−1^ against A-549 cancer cells [37]. In addition, Elkomy et al. [38] also reported the anticancer activity of *Oscillatoria simplicissima* against the A-549 cancer cell line with 49.465% cell viability. The authors confirmed the results using an SBR assay. Lyngbyabellins are a type of peptolide found in various marine cyanobacteria genera, including *Moorea* and *Okeanea* [39]. Lyngbyabellins have been reported to possess anticancer properties against various cancer cell lines [40]. A study isolated ten lyngbyabellin compounds (lyngbyabellin G, lyngbyabellin O, lyngbyabellin P, lyngbyabellin H, 27-deoxylyngbyabellin A, R=H, homohydroxydolabellin, and lyngbyabellin A, R=OH) from Malaysian *Moorea bouillonii* and Red Sea *Okeania* sp., and the isolated compounds were tested for their cytotoxic activity against human breast cancer cells (MCF7). All the tested lyngbyabellin compounds exhibited varying levels of cytotoxic activity based on their structure [39]. Furthermore, Caldorin is a cytotoxic polyketide found in marine cyanobacteria *Caldora penicillate*. It was reported that Caldorin showed moderate or weak cytotoxic activity against the HeLa cell line and HL 60 cell line, which are commonly used in cancer research [41]. A study was conducted to isolate the aplysia toxin derivatives, neo-debromoaplysia toxin I, and neo-debromoaplysiatoxin J from the marine cyanobacterium *Lyngbya* sp. The cytotoxicity of these compounds was assessed using the SW480 human colorectal carcinoma cell line, SGC7901 human gastric cancer cells, LoVo human colorectal carcinoma cells, and PC-9 non-small-cell lung cancer cells. According to the obtained data, neo-debromoaplysiatoxin I resulted in weak cytotoxic activity while neo-debromoaplysiatoxin J exhibited strong cytotoxic activity against the tested cancer cell lines, with less than 20% cell viability [42]. The marine cyanobacterium *Lyngbya* sp. was reported to contain chlorinated leucine-derived natural compounds. These compounds exhibit structural similarity to polychlorinated leucine, which is known to possess a range of bioactivities including anti-cancer activity [43].

The above studies suggest that marine cyanobacteria could be a potential source of natural products with anticancer properties. The extracts and compounds from marine cyanobacteria exhibit significant anticancer activity against various cancer cell lines, indicating their potential for development of novel anticancer drugs with fewer side effects than conventional chemotherapy drugs. However, further research is required to identify and isolate new bioactive compounds from marine cyanobacteria and understand their mechanisms of action. Therefore, more studies are needed to explore the full potential of marine cyanobacteria as a source of anticancer agents.

### 3.3. Hepatoprotective Properties

The liver is a crucial organ in the human body, performing vital functions in metabolism and detoxification. Due to the importance of these functions, hepatic diseases pose a serious threat to people’s health, leading to significant health complications [44]. Despite the advances of modern medicine, there is still no effective therapeutic agent that can stimulate liver function, prevent liver damage, and help regenerate hepatic cells. However, C-Phycocyanin (C-PC) isolated from the cyanobacterium *Phormidium versicolor* has been shown to have hepatoprotective activity against cadmium-induced liver injury in rats. Cadmium is an industrial pollutant that increases the production of reactive oxygen species (ROS) in liver tissues and lipid peroxidation. Liver damage caused by cadmium is indicated by the induced levels of hepatic serum enzymes such as alanine transaminase (ALAT), aspartate transaminase, and total bilirubin. Phycocyanin treatment resulted in decreased levels of ALAT, ASAT, and bilirubin in rats with cadmium-induced liver injury. Cadmium suppresses radical scavenging enzymes like superoxide dismutase (SOD), catalase (CAT), and glutathione peroxidase (GPx), leading to an increase in ROS production. The findings of this study demonstrated an increase in the levels of SOD, CAT, and GPx in phycocyanin-treated rats compared to cadmium-treated rats [17]. Another study revealed that phycocyanin isolated from *S. platensis* was found to have hepatoprotective activity against carbon tetrachloride (CCL_4_)-treated Albino rats. The hepatoprotective effect of phycocyanin was determined by investigating the activities of the liver enzymes alanine transaminase (ALT), aspartate transaminase (AST), creatinine, and urea. ALT enzyme indicates cell membrane damage while AST indicates mitochondrial damage. According to the results, rats injected with a phycocyanin concentration of 200 mg/Kg showed the highest reduction in ALT, AST, creatinine, and urea concentration compared to the positive control [45]. Furthermore, it was observed that *S. platensis* reduced oxidative stress induced by CCL4 in male rats by enhancing the activities of antioxidant enzymes, including GPx, SOD, CAT, and glutathione content, while inhibiting lipid peroxidation products and nitric oxide levels in the rat liver. Furthermore, *S. platensis* was found to counteract the increased hepatic levels of Ki-67, interleukin-6, tumor necrosis factor-alpha (TNF-α), and COX-2 messenger RNA expression induced by CCL4. Additionally, reduced p53 expression levels were observed in the *S. platensis* + CCL4-treated rats. The study revealed that *S. platensis* can cause the cell death of injured hepatocytes and prevent liver dysfunction caused by CCL4 [46].

### 3.4. Antidiabetic Properties

Diabetes mellitus is one of the concerning health issues which affects people of different age groups worldwide. It is a type of metabolic disorder in which a person has a high blood glucose level over some time due to insufficiency in insulin secretion or insufficiency in insulin activity of the body [47]. The antidiabetic activity of marine cyanobacteria makes them useful as potential antidiabetic sources for antidiabetic drugs. Phycocyanin, a water-soluble protein isolated from *S. platensis*, showed antidiabetic activity through α-amylase and β-glucosidase enzyme inhibition. The β-glucosidase enzyme activity of phycocyanin from *S. platensis* was reported to have increased activity with the increased concentration of phycocyanin. The α-amylase enzyme activity of phycocyanin from *S. platensis* was also reported to have increased activity with the increased concentration of phycocyanin [11]. According to a study conducted by Ahmed et al. [48], *Fischerella* sp. BS1-EG proved to have anti-diabetic activity by potential inhibition of α-glucosidase by 7.56%. Υ-Aminobutyric acid (GABA) is a bioactive compound present in both eukaryotes and prokaryotic organisms. Due to the important physiological functions of GABA, it aids in preventing and controlling various diseases, including diabetes [48]. Cyanobacteria were reported to produce GABA as a survival strategy against environmental stresses. A recent study evaluated Irish marine cyanobacteria as an alternative source for GABA production. In this study, seventeen Irish marine cyanobacteria species were screened as potential GABA producers. Among them, twelve cyanobacteria species tested positive for GAD (Glutamate decarboxylase) activity in vitro. Among the twelve cyanobacteria species that showed GAD activity, five cyanobacteria species exhibited a characteristic GABA peak in the spectrophotometric assay, indicating the production of GABA [49].

### 3.5. Anti-Aging Capabilities

Aging is an inevitable biological process that affects everyone, and skin aging is a complex phenomenon that is associated with both age-dependent and premature aging. While age-dependent aging is an intrinsic process, premature aging is caused by extrinsic factors such as changes in the environment, diet, exposure to sunlight, and pollution [50]. Skin dryness, wrinkles, and decreased elasticity are the main aging expressions. The inhibition of hyaluronidase, elastase, and collagenase enzymes leads to the decrease of wrinkles and the enhancement of skin elasticity [14]. According to a study conducted by Pagels et al. [14], two pigment-targeted extracts, rich in carotenoids and phycobiliproteins, from the marine cyanobacterium *Cyanobium* sp. were screened for hyaluronidase, tyrosinase, elastase, and collagenase activities. The results showed that the carotenoid-targeted extract was able to inhibit the hyaluronidase enzyme activity while the phycobiliprotein-targeted extract was able to inhibit the hyaluronidase and collagenase enzyme activities. In another study, *Cyanobium* sp. LEGE07175 and *Tychonema* sp. LEGE 07196 were screened for hyaluronidase inhibition. Both species showed hyaluronidase inhibition potential. However, among those two species, *Tychonema* sp. LEGE 07196 resulted in the highest inhibitory activity, with an IC_50_ value of 182.74 μgmL^−1^, while *Cyanobium* sp. LEGE 07175 resulted in an IC_50_ value of 208.36 μgmL^−1^ [51]. According to Nowruzi et al. [52], protein-rich Spirulina extract has been identified as a potential source for the development of anti-aging products. Moreover, *Chlorogloeopsis* sp. exhibited anti-aging properties by preventing the formation of free radicals in the presence of UV-A and UV-B radiation. Another review article by Nowruzi et al. [52] reported that *Chlorogloeopsis* sp. exhibited anti-aging properties by preventing the formation of free radicals in the presence of UV-A and UV-B radiation. Overall, the review by Nowruzi et al. [15] highlights the potential of natural sources, such as Spirulina extract and *Chlorogloeopsis* sp., in the development of anti-aging products. The extracellular matrix (ECM) is the largest component of the dermal skin, which constitute over 70% of the skin. It is composed of collagen and glycosaminoglycans covalently linked to proteins, forming the proteoglycans. The human skin contains different types of collagenase enzymes which initiate collagen fragmentation and collagen turnover by cleaving all types of collagen present in the human skin. This cleavage ultimately results in loss of dermal homeostasis and tissue damage. Several cyanobacteria were reported to have compounds that can inhibit the enzymes responsible for the digestion of extracellular matrix compounds. A study performed with the cyanobacterium *Aphanothece halophytica* demonstrated that it contains Mycosporine-2-glycine (M2G) a type of mycosporine-like amino acid (MAA) which has collagenase-inhibitory activity. In this study, M2G showed collagenase-inhibitory activity (Table 1), with an IC_50_ value of 0.47 mmol^−1^, making the cyanobacterium *Aphanothece halophytica* a potential source of anti-aging compounds [15].

### 3.6. Anti-Obesity Potential

Obesity is one of the concerning metabolic diseases at present. It can increase the risk of many health conditions including type II diabetes, cardiovascular diseases, osteoarthritis, and cancer of the esophagus, colon, rectum, liver, gallbladder, pancreas, and kidney [53]. Obesity is caused by the imbalance between energy intake and energy expenditure. This imbalance is caused by the alteration of lipogenesis and lipolysis. Modulation of the intake of dietary lipids triacylglycerol and cholesterol and phospholipids is an effective way to treat obesity. A group of researchers screened 263 cyanobacterial fractions for PED6 activity using Zebrafish larvae as an in vivo animal model [54]. In total, 11 out of 263 cyanobacterial fractions reduced PED6 activity by 30%–40%. The reduction of PED6 activity is related to the inhibition of intestinal phospholipid activity and gastrointestinal phospholipid absorption. Yoshinone A is a bioactive molecule derived from cyanobacterium *Leptolyngbys* sp. Yoshinone A is reported to inhibit the adipogenic differentiation in the mouse fibroblastic cell line 3T3-L1 [16].

Chlorophylls are some of the most abundant photosynthetic pigments present in marine cyanobacteria. Chlorophyll a is the major type of chlorophyll found in cyanobacteria, while some cyanobacteria species have been reported to possess additional chlorophylls, such as Chlorophyll d and Chlorophyll f [55]. Chlorophylls derived from cyanobacteria could be structurally altered into various derivatives: pheophorbides, pheophytin, porphycene, porphyrins, and phthalocyanines [56,57]. Freitas et al. [56] investigated the presence of chlorophyll derivatives in the cyanobacteria *Cyanobium* sp. LEGE 07175 and *Nodosilinea* sp. LEGE 06001. The chlorophyll derivatives 13^2^-hydroxy-pheophytin and 13^2^-hydroxy-pheofarnesin were successfully isolated. The isolated chlorophyll derivatives were tested for lipid-reducing activity using a Zebrafish Nile red fat metabolism assay. 13^2^-hydroxypheophytin and 13^2^-hydroxy-pheofarnesin derivatives effectively reduced the Nile red staining, with half the maximum effective concentration (EC_50_) concentrations of 8.9 ± 0.4 μM and 15.5 ± 1.3 μM. Furthermore, both derivatives reduced neutral lipid accumulation in 3T3-L1 multicellular spheroids of murine preadipocytes.

### 3.7. Neuroprotective Activity

**Figure 2:**
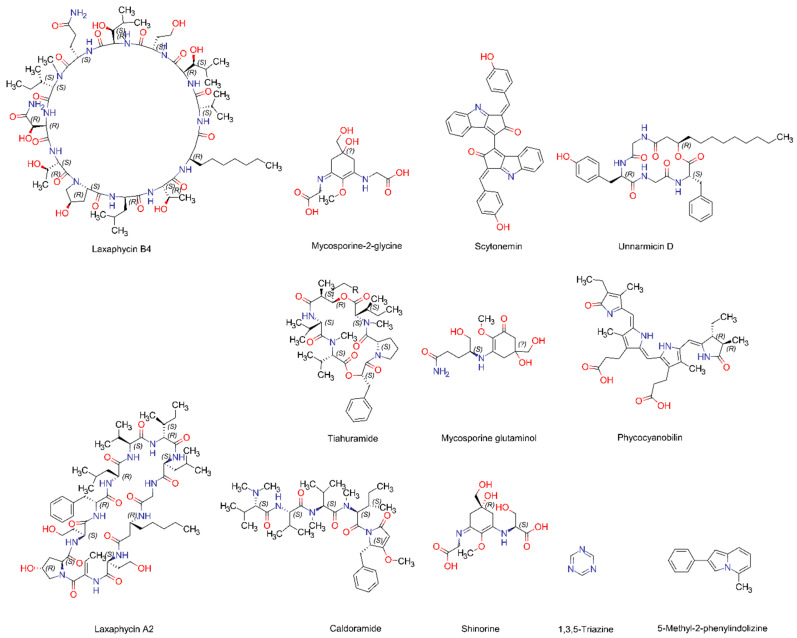
Chemical structures of bioactive compounds isolated from marine cyanobacteria.

Neurodegenerative diseases affect millions of people around the world. The World Health Organization predicts neurodegenerative diseases will be the second leading cause of death by 2040 [58]. Most of the neurons originate in the brain and they can be found throughout the body. The proper functioning of neurons is most important as they play a critical role in human brain activity. Neurodegeneration is associated with the continuous loss of neurons, neuron structure, or its functions [59]. Neurodegeneration also causes abnormalities in the brain and spinal cord, causing muscle weakness, permanent paralysis, and dementia. Common neurodegenerative diseases include Alzheimer’s disease (AD), Parkinson’s disease, Huntington’s disease, and prion disease [59,60]. Acetylcholinesterase enzyme (AChE) is the targeted molecule for drug use in the treatment of AD. Acetylcholinesterase enzyme (AChE) inhibits the Acetylcholine neurotransmitter, which is essential for processing memory and learning. Patients with AD have a deficiency in Acetylcholine due to the activity of the Acetylcholinesterase enzyme (AChE). Since the synthetic drugs which are used to inhibit the activity of the Acetylcholinesterase enzyme (AChE) have adverse side effects, researchers are now focusing on compounds extracted from natural resources. A study was conducted to assess the AChE inhibitory potential of the cyanobacterium *Oscillatoria sancta*. According to the obtained results, *O. sancta* extracts greatly reduced AChE activity, suggesting their potential use in treating Alzheimer’s disease [61]. One of the distinctive features of Alzheimer’s disease (AD) is the deposition of insoluble aggregates of amyloid β-peptide (Aβ) in the brain and its blood vessels [62,63]. In a study conducted by Koh et al. [62], *Spirulina maxima* 70% ethanol extract was tested for its neuroprotective effects against Aβ-induced neurotoxicity in PC12 cells. According to the data obtained from this study, *S. maxima* was able to cause cell death via the activation of BDNF (Brain-Derived Neurotrophic Factor) signaling [62]. Glutamate is a neurotransmitter that can be found in the central nervous system. When an excessive amount of glutamate is present, it causes neuronal cell death, which is a major causative factor for neurodegenerative diseases. Lee et al. [64] investigated the neuroprotective effect of water-extracted *Spirulina maxima* on glutamate-induced neuronal cell death in mouse hippocampal HT22 cells. The study reported that the 10 and 100 μg/mL concentrations of *S. maxima* have the potential to reduce glutamate-induced cell death in HT22 cells. In another study, the neuroprotective activities of fermented *S. maxima* on glutamate-treated HT22 cells were investigated. According to the results, β-carotene-containing *S. maxima* extract had the highest neuroprotective activity (Table 1), with a rate of 82.96% [62]. Additionally, three new cyclic depsipeptides, Tiahuramides A, B, and C (Figure 2), were isolated from a French Polynesian collection of the marine cyanobacterium *Lyngbya majuscule*. The cytotoxic activity of these compounds was evaluated on a human neuroblastoma SH-SY5Y cell line using an MTT assay. The study reported that Tiahuramides B and C demonstrated IC_50_ values of 14 and 6 μM, respectively. These findings suggest that Tiahuramides B and C may have the potential to act as therapeutic agents [65].

### 3.8. Antioxidant Activity

Oxidative stress refers to the imbalance between the production and degradation of reactive oxygen species (ROS) or reactive nitrogen species (RNS). Oxidative stress has been implicated as one of the main causative factors in the development of chronic and degenerative diseases, such as aging, diabetes, arthritis, cardiovascular diseases, and cancer [66]. Photosynthetic organisms like cyanobacteria have various approaches to prevent damage caused by reactive oxygen species (ROS). Cyanobacteria contain a wide variety of pigments that have antioxidant properties. Phycocyanin is a blue-colored pigment that is abundant in cyanobacteria. In a study conducted by Renugadevi et al. [67], the phycocyanin pigment that was extracted from the filamentous cyanobacteria *Geitlerinema* sp. was tested for its antioxidant properties. According to the results, the DPPH radical-scavenging effect of the extracted pigment was found to be the highest at a concentration of 200 μg/mL, with a scavenging activity of 78.75%. Ferric-reducing antioxidant activity of the extracted pigment was also observed at a concentration of 200 μg/mL. Furthermore, the maximum hydrogen-peroxide-radical-scavenging activity of the extracted phycocyanin was recorded as 95.27% at the same concentration. The maximum anti-lipid peroxidation activity of phycocyanin was observed to be 53.65% at 200 μg/mL [67]. In another study, Phycoerythrin (Figure 2) isolated from *Halomicronema* sp. R31DM was screened for antioxidant activity in vitro and in vivo. According to the results, Phycoerythrin isolated from *Halomicronema* sp. R31DM had dose-dependent DPPH scavenging activity of up to 64% at a 100 μg dose. In addition, the in vivo antioxidant activities of Phycoerythrin was also tested using *Caenorhabditis elegans* worms and found to have promising antioxidant properties under the tested conditions (oxidative stress resistance assay, thermal stress resistance assay, lifespan assay, and pharyngeal) [68]. Moreover, a study conducted by Konstantinou et al. [13] found it to have antioxidant activity in *Leptothoe* strains (TAU-MAC 0915, 1015, 1115, and 1215) [69]. The DPPH radical-scavenging activity of C-phycocyanin in Phycobiliprotein extracted from *Arthrospira platensis* resulted in 54% inhibition at 0.02 g/mL concentration. Another study was conducted to assess the antioxidant activity of crude polysaccharides extracted from the marine cyanobacteria *Oscillatoria simplicissima* and *O. acutissima*. The results showed that the highest percentage of inhibition of 45.97% was in crude polysaccharides extracted from *O. simplicissima*, while it was 42% in crude polysaccharides extracted from *O. acutissima*. In another study, the antioxidant activity of different solvent extracts (dH2O, ethanol: dH2O(1:1), ethanol: dH2O(4:1), and ethanol) of the cyanobacteria *Scytonema* sp. and *Lyngbya* sp was tested using DPPH and ABTS assays. According to the results, solvent extracts (dH2O and ethanol: dH2O(4:1)) of *Scytonema* sp. showed the highest activity with the ABTS assay while solvent extracts (ethanol: dH2O(4:1)) of *Lyngbya* sp presented the highest activity with the ABTS assay. The aqueous extract of *Scytonema* sp. showed the highest activity with the DPPH assay while solvent extracts (ethanol: dH2O(4:1)) of *Lyngbya* sp presented the highest activity with the DPPH assay [19]. Mycosporine-like amino acids (MAAs) are multifunctional secondary metabolites synthesized by cyanobacteria. MAAs are known to have antioxidant properties. A study was conducted to screen a total 53 Irish marine cyanobacteria species in order to identify potential producers of MAAs. LC-MS analysis revealed the presence of eight types of known MAAs in eight cyanobacteria species. All of these MAAs exhibited antioxidant activities through DPPH, FRAP, and ORAC assays [70]. In a separate study, the marine cyanobacteria *Lyngbya majuscule* SB 12-13 and *Lyngbya martensiana* SBD24, isolated from the Odisha coast, India, were tested for antioxidant activity using DPPH and ABTS radical-scavenging assays. Between the two species, the highest antioxidant activity was recorded in *Lyngbya majuscule* [71].

### 3.9. Antimicrobial Activity

In the last few decades, infectious diseases have increased to a great extent, affecting public health. Antibiotic resistivity has become a major therapeutic problem in recent years. Marine cyanobacteria are known to have compounds with antibacterial activity. Cyanobacteria-derived natural peptides were reported to have antibiotic activity. These peptide antibiotics are categorized into four groups; depsipeptides, lipopeptides, cyclamides, and cyclic peptides [72]. Three new cyclic depsipeptides, tiahuramides A, B, and C, from a French Polynesian collection of the marine cyanobacterium Lyngbya majuscule were tested for antibacterial activity against three opportunistic marine pathogenic bacteria, *Aeromonas salmonicida*, *Vibrio anguillarum*, and *Shewanella baltica*. Tiahuramide C (Figure 2) was reported to have the highest antibacterial activity with Minimum Inhibitory Concentration (MIC) values of 7, 7, and 16 μM against *A. salmonicida*, *V. anguillarum*, and *S. baltica*, respectively. Moreover, Tiahuramide B showed the highest antibacterial activity with MIC values of 12 and 29 μM against Gram-negative (*Escherichia coli*) and Gram-positive (*Micrococcus luteus*) bacteria compared to tiahuramides A and C [65]. A study conducted by Elkomy et al. [73] screened marine cyanobacteria *O. simplicissima*, *O. acutissima*, and *S. platensis* for antibacterial activity against pathogenic Gram-negative and Gram-positive bacteria. The data showed that the methanol extract of three cyanobacteria had more activity against most of the pathogenic bacteria (*Staphylococcus aureus*, *M. luteus*, *Serratia marcescene*, *Salmonella* spp., *Vibrio* spp., *Aeromonas hydrophila*, *Pseudomonas aeruginosa*, and *E. coli*). Organic solvents extracts (Chloroform, Acetone, Dichloromethane, and Ethyl acetate) of marine cyanobacteria Oxynema thaianum ALU PBC1, PBC2, PBC3, PBC4, PBC5, PBC6, PBC7, and PBC8 was tested for antibacterial activity against *E. coli* and *Klebsiella pneumoniae* by disk diffusion assay. Among all the extracts tested, chloroform extract of *O. thaianum* ALU PBC5 showed effective zones of clearance against *E. coli* and *K. pneumoniae*. Hence, *O. thaianum* ALU PBC5 can be used as a promising source to treat multi-drug-resistant pathogens. The marine cyanobacterium *Oscillatoria* sp., isolated from the coastal region of west Malaysia, was tested for antibacterial activity against *S. aureus*, *B. subtilis*, *E. coli*, and *P. aeruginosa* by a well diffusion assay. According to the data, crude extract of *Oscillatoria* sp. effectively inhibited the growth of *S. aureus*, *Bacillus subtilis*, *E. coli*, and *P. aeruginosa* at the concentrations of 100 mg/mL. Moreover, the methanolic extract of *Oscillatoria* sp. effectively inhibited the growth of *S. aureus* and *P. aeruginosa* at MIC values of 30 and 25 μg/mL, respectively. The Gas chromatography-Mass Spectroscopy analysis of *Oscillatoria* sp. extract identified antimicrobial compounds such as 1,3,5-triazine, 5-Nitro-3-cyano-2(1H)-pyridone, aceticacid, and 5-methyl-2-phenyl indolizine [74]. A study conducted by Grubisic et al. [75] screened the marine cyanobacterium *Euhalothece* sp. C1 for antibacterial ad antifungal activity against bacteria (*E. coli*, *B. subtilis*, *S. aureus*, *P. aeruginosa*, and *Enterococcus faecalis*), a fungus (*Aspergillus niger*), and yeast (*Candida utilis*) using a disk diffusion assay. Among these, the methanolic extract of *Euhalothece* sp. C1 showed an inhibitory effect against *E. coli*, *P. aeruginosa*, *S. aureus*, *E. faecalis*, and *C. utilis*. Ethanol extracts of the marine cyanobacterium *Rivularia mesenterica* were also reported to have antibacterial and antifungal activity against *B. cereus*, *Clostridium perfringens*, *M. luteus*, *S. aureus*, *Enterobacter sakazakii*, *E. coli*, *Enterobacter cloacae*, *K. pneumoniae*, *P. aeruginosa*, *A. niger*, *Candida albicans*, *Penicillium* sp., and *Saccharomyces cerevisiae* at MIC values in the range 0.06 to 32.00 μg/mL [76]. Furthermore, the cyanobacteria *Stigonema* sp. and *Spirulina* sp., isolated from the southeast coast of India, were screened for antibacterial and antifungal activity against the bacteria *Bacillus* sp., *K. pnemoniae*, *Protease* sp., *P. aeruginosa*, and *S. aureus* and the fungi *Aspergillus flavus*, *Aspergillus fumigatus*, *Aspergillus ochraceus*, *Aspergillus terreus*, and *Trichoderma viride* using the agar well diffusion method. Methanol and hexane extracts of *Stigonema* sp. showed effective inhibition at a concentration of 100 μL, while methanol and hexane extracts of *Spirulina* sp. showed effective inhibition at a concentration of 100 μL [77]. The *Oscillatoria sancta* marine cyanobacterium was reported to show antibacterial activity against *Bacillus cerus* under starvation conditions [61].

### 3.10. Photoprotective Properties

Solar ultraviolet radiation (UV-R) compromises three categories, depending on wavelength; UV-A radiation (320–400 nm), UV-B radiation (280–320 nm), and UV-C radiation (200–280 nm). Among these, UV-B radiation is considered the most harmful since it induces gene mutations in skin cells. Additionally, UV-A radiation also induces gene mutations indirectly by producing reactive oxygen species. Therefore, to protect the human skin from these harmful UV radiations, synthetic sunscreens are being used. However synthetic sunscreen is often associated with several negative impacts, for instance, allergic reactions, photo-toxicity, and hormone disorders. As a solution to this problem, scientists are now focusing on natural photoprotective alternatives. Due to the photoautotrophic nature of cyanobacteria, they have adapted to protect themselves from harmful UV radiation. One of the main adaptations of cyanobacteria is the production of UVR-absorbing compounds such as scytonemin and mycosporine-like amino acids (MAA). An oil–water cream that contains hydroethanolic extract of the cyanobacterium *Scytonema* sp. was screened in vitro for its photoprotective capacity. According to the results of the study, the highest absorption increase showed in the UV-B region (between 309–312 nm) was by 15% in comparison with the base cream. In concordance with the calculated indexes, the sun protection factor (SPF) was recorded as 1.9. In this study, among the aqueous extracts of cyanobacteria, the highest level of scytonemin was detected in *Scytonema* sp., followed by *Lyngbya* sp. Mycosporine-glutaminol was identified for the first time in *Scytonema* sp., showing a peak in the UV-B region (310–312 nm) [19]. Shinorine is a mycosporine-like amino acid (MAA) that has strong UV absorption properties. Joshi et al. [78] discovered shinorine from *Leptolyngbya* sp. isolated from Vagator beach, Goa, India. Based on the UV/vis absorption spectra and Electrospray-ionization liquid chromatography-mass spectrometry (ESI-LCMS) analysis of crude methanol extract of *Leptolyngbya* sp., shinorine was identified.

## 4. Conclusions

According to the published literature, marine cyanobacteria have been reported to possess various bioactive properties. Although extracts obtained from cyanobacteria have demonstrated bioactivity in certain studies, the structure of the bioactive compounds responsible for these observed activities remains unclear. Therefore, further research is needed to gain a better understanding of the structure and functional groups associated with the bioactive compounds present in marine cyanobacteria species.

Currently, most studies in the literature focus on pre-clinical investigations. Since only a limited number of clinical studies have been conducted to assess the bioactive properties of marine cyanobacteria, it is recommended to conduct more extensive animal studies and well-controlled clinical trials for further research. These studies will provide essential information on the efficacy and possible side effects of the identified bioactive compounds derived from marine cyanobacteria.

In conclusion, there is significant potential for the development of new therapeutics utilizing marine cyanobacteria to combat human diseases and safeguard human health. However, further research is necessary to elucidate the structure–function relationship of bioactive compounds, conduct more clinical studies, and assess the safety and efficacy of these compounds.

**Table 1:** Compounds with bioactive properties identified from cyanobacteria that are beneficial for human health.

Compounds	Cyanobacteria Species	Bioactive Properties	IC50/MIC/EC50/Cell Line/Target Bacteria/% of Inhibition	References
Glycolipids Phospholipids	*Spirulina subsalsa*	Anti-inflammatory	ND	[7]
Peptides	*Synechococcus* sp.	Anti-inflammatory	LPS-stimulated RAW 264.7 macrophage cells	[79]
Xanthophyll	*Leptolyngbya-like* sp. LEGE13412	Anti-inflammatory	ND	[27]
Unnarmicin D	*Trichodesmium thiebautti*,	Anti-inflammatory	Murine BV-2 cells	[30]
Laxaphycins B4 Laxaphycins A2	*Hormothamnion enteromorphoids*	Anticancer	IC50;1.7μM IC50;2μM HCT116 cells	[33]
Caldoramide	*Caldora penicillata*	Anticancer	HT-29,HCT116 and MCF-7 cell lines.	[35]
Phycocyanin	*Spirulina platensis* *Phormidium versicolor* *Spirulina maxima* *Arthrospira platensis* *Geitlerinema* sp.	Anticancer Hepatoprotective Antidiabetic Neuroprotective Antioxidant	Vero cell lines and Hep-G2 cell lines. 78.75% (DPPH)	[11,13,17,45,62,64,67]
Carotenoids phycobiliproteins	*Cyanobium* sp.	Antiaging	ND	[14]
Mycosporine-2-glycine (M2G)	*Aphanothece halophytica*	Antiaging	IC50; 0.47 mmol^−1^	[15]
Yoshinone A	*Leptolyngbys* sp.	Anti-obesity	3T3-L1	[16]
13^2^-hydroxy-pheophytin	*Cyanobium* sp. LEGE 07175	Anti-obesity	EC50; 8.9 ± 0.4 μM	[56]
13^2^-hydroxpheofarnesin	*Nodosilinea* sp. LEGE 06001	Anti-obesity	EC50; 15.5 ± 1.3 μM	[56]
Chlorophyll a	*Spirulina maxima*	Neuroprotective	PC12 cells	[62]
β-carotene	*Spirulina maxima*	Neuroprotective	HT22 cells	[62]
Tiahuramides B	*Lyngbya majuscule*	Neuroprotective Antibacterial	IC50; 14 μM (SH-SY5Y cell line) MIC; 9.4, 8.5 μM (*A. salmonicida*, *V. anguillarum*) MIC; 12, 29 μM (*Escherichia coli*, *Micrococcus luteus*)	[65]
Tiahuramides C		Neuroprotective Antibacterial	IC50; 6 μM (SH-SY5Y cell line) MIC; 7, 7, 16 μM (*A. salmonicida*, *V. anguillarum*, *S. baltica)*	
Tiahuramides A		Antibacterial	MIC; 27, 33 μM (*A. salmonicida*, *V. anguillarum*)	
Phycoerythrin (PE)	*Halomicronema* sp. R31DM	Antioxidant	ND	[68]
Polysaccharides	*Oscillatoria simplicissima*	Antioxidant Anticancer	45.97% (DPPH) A-549 cell line	[38]
Myc-glutaminol Palythine Asterina 330	*Scytonema* sp. *Lyngbya* sp.	Antioxidant Photoprotective	ND	[19]
1,3,5-triazine, 5-Nitro-3-cyano-2(1H)-pyridone acetic acid 5-methyl-2-phenyl indolizine	*Oscillatoria* sp.	Antimicrobial	ND	[74]
scytonemin	*Scytonema* sp. *Lyngbya* sp.	Photoprotective	ND	[19]
Shinorine	*Leptolyngbya* sp.	Photoprotective	ND	[78]

## Data Availability

Not applicable.
